# Urinary metabolites as a predictive marker for perinatal depression: A secondary analysis of the mothers, Omega-3 & Mental Health Study

**DOI:** 10.1016/j.psycom.2022.100046

**Published:** 2022-04-30

**Authors:** Patricia S. Greco, Ashley M. Hesson, Ellen Mozurkewich, Deborah R. Berman

**Affiliations:** a University of Michigan, Department of Obstetrics and Gynecology, United States; b University of New Mexico, Department of Obstetrics and Gynecology, United States

**Keywords:** Perinatal, Postpartum, Depression, Screening, Metabolomics

## Abstract

**Background:**

Perinatal depression has been associated with unfavorable pregnancy and childhood development outcomes; however, no objective markers exist to identify perinatal mood disorders. We investigated whether metabolites in maternal urine during pregnancy can predict increased depressive symptoms in late pregnancy and postpartum among pregnant women at risk for perinatal depression.

**Methods:**

We evaluated metabolomic markers in urine collected at 12–20 and 34–36 weeks’ gestation. We analyzed 49 urinary metabolites using ion pairing reversed-phase liquid chromatography-mass spectrometry. Depressive symptom severity was identified using Beck Depression Inventory (BDI) scores from 105 participants at 12–20 and 34–36 weeks’ gestation, and 6–8 weeks’ postpartum. Mixed model repeated measures analysis evaluated associations between changes in maternal urinary metabolites and BDI scores across pregnancy.

**Results:**

Increases in urinary xanthine and hypoxanthine were positively associated with increases in maternal depressive symptoms throughout pregnancy (*p* = 0.03 and 0.02, respectively). This finding did not persist after false discovery rate correction. None of the urinary metabolites examined were significantly associated with development of postpartum depressive symptoms.

**Limitations:**

This study is an exploratory secondary biologic sample analysis from a trial whose sample size was determined by a different primary outcome and expected effect size, which may have limited statistical power to detect associations between urinary metabolites, depressive symptoms, and mood trajectory over time.

**Conclusions:**

Increasing concentrations of xanthine and hypoxanthine were associated with increasing depressive symptoms throughout pregnancy. Further research is needed to evaluate the utility of these metabolic markers in identifying women at risk for perinatal depressive symptoms.

## 1. Introduction

Major Depressive Disorder (MDD) is prevalent among women of childbearing age, with 20–25% of women being affected at least once during their lifetime (Kessler et al., 2003). Perinatal depression is a mood disorder that encompasses both prenatal depression and postpartum depression (Woody et al., 2017). Nearly 40% of women with MDD experience depression for the first time after giving birth, which is also known as postpartum depression (PPD) (Wisner et al., 2013). More recently, prenatal depression has been reported to have an even higher prevalence than PPD (Woody et al., 2017). It has been theorized that interactions between hormones may play an important role in predisposing women to developing MDD; hence, pregnant and postpartum women are vulnerable to MDD due to significant hormonal fluctuations (Solomon and Herman, 2009). Studies suggest that low levels of progesterone and/or estrogen, as seen during the peripartum period, can increase the risk of developing depression or depressive symptoms (Brummelte and Galea, 2010). Furthermore, dysregulation of the hypothalamic pituitary adrenal (HPA) axis has been found to be the most well-known hormonal cause of depression (Lightman et al., 2001). The HPA axis produces stress hormones, such as cortisol. The reactivity of the HPA axis is altered by the changes in steroid hormones during pregnancy (Brummelte and Galea, 2010). Not only has depression been associated with altered levels of several stress hormones, but depressive symptoms in pregnancy have also been associated with changes in inflammatory markers and HPA axis hormones (Nazzari et al., 2020). With a 10–15% incidence in all deliveries, depression is one of the most common complications of pregnancy (Wisner et al., 2013).

Maternal depression during a pregnancy has been shown to be a risk factor for unfavorable pregnancy outcomes including substance use disorder, poor weight gain, lack of prenatal care, prematurity, lower infant birth weight, and decreased Apgar scores at birth (Brummelte and Galea, 2016; Miller, 1991; Steer et al., 1992). Perinatal depression has been found to affect not only maternal morbidity and mortality, but also maternal-infant interactions, and may be related to adverse child outcomes due to disrupted neurobiological development (Kelly et al., 2002). Research has illustrated that the emotional development of the child is affected by both *in utero* biological programming of fetal development, as well as maternal caregiving behavior (Glover et al., 2008). The primary mechanism believed to be involved in *in utero* biological fetal programming is exposure to maternal cortisol. Elevated levels of maternal stress hormones impact the developing fetal HPA axis, thus affecting neonatal neurobiological, immune, and metabolic development (Cao-Lei et al., 2016). Lastly, it has been shown that depression is negatively associated with IQ and language development in the child (Previti et al., 2014).

Diagnosis of MDD is currently made using the Diagnostic and Statistical Manual of Mental Disorders (DSM-5) via subjectively assessed criteria (American Psychiatric Association, 2013). The Edinburgh Postnatal Depression Scale (EPDS) is a subjective tool used to assist in the identification of at-risk women prior to diagnosis (Cox et al., 1987). Additionally, the Beck Depression Inventory (BDI) may be used to assess depressive symptom severity (Beck et al., 1961, 1988). No objective serologic or biochemical methods to diagnose perinatal depression (either prenatal or PPD) exist.

Metabolomics, which can measure molecular concentrations within bio-samples, has been used extensively to characterize metabolic changes in diseases. Therefore, it is theorized that metabolomics can help identify various disease processes ranging from mental illnesses, such as schizophrenia, to oncologic diagnoses, such as bladder cancers (Cheng et al., 2010; Huang et al., 2011; Nicholson and Lindon, 2008). More recently, Zheng et al. and Lin et al. studied urinary metabolite markers in women with diagnoses of MDD and PPD, respectively (Lin et al., 2017; Zheng et al., 2013).

Zheng et al. identified a panel consisting of five urinary metabolites—malonate, formate, N-methyl-nicotinamide, m-hydroxyphenylacetate, and alanine—as potential candidates for development of a laboratory-based test for detection of MDD (Zheng et al., 2013). Similarly, Lin et al. identified 22 urinary markers that correlated with development of PPD. They identified a more specific panel consisting of five urinary metabolites—formate, succinate, dimethylamine, α-glucose, and 1-methylhistidine—that were distinguishable from subjects with PPD compared to those who were postpartum without a diagnosis of PPD (Lin et al., 2017). In Zheng et al.‘s work, the subjects were all recruited, and the specimens collected, after they had a known diagnosis of MDD. In Lin et al., the subjects, and thus the specimens, were recruited and collected after having also received a diagnosis of PPD.

Researchers have not yet explored metabolomic markers that may predict the onset of depression or those who are at risk for depressive symptoms. Urine samples are an easy biological specimen to obtain and study, making them an excellent target for evaluation of metabolomic markers. Prior exploratory studies have evaluated urinary metabolites in patients diagnosed with depression; thus, urine samples were an excellent source for this study (Lin et al., 2017; Zheng et al., 2013). The objective of this study was to determine whether metabolic components of maternal urine samples during pregnancy can be used to predict increased depressive symptoms in late pregnancy and postpartum among pregnant women at risk for perinatal depression. We hypothesized that stress hormone-related urinary metabolites from early and late pregnancy samples would be significantly associated with depressive symptoms in late pregnancy or postpartum.

## 2. Methods

This study was an exploratory secondary analysis of urine samples collected for the Mothers, Omega-3 & Mental Health Study, a double blind, placebo-controlled randomized controlled trial of eicosapentaenoic acid (EPA)- and docosahexaenoic acid (DHA)-rich fish oils for prevention of depressive symptoms among women at risk for depression that was carried out between October 2008 and January 2012.

The original trial was registered at clinicaltrials.gov (NCT00711971) under the title “Does Fish Oil Prevent Depression in Pregnancy and Postpartum?” and was approved and conducted in accordance with the institutional review boards of the University of Michigan, study number HUM00004684; and St. Joseph Mercy Hospital in Ypsilanti, Michigan. All subjects gave written, informed consent to participate in the study (Mozurkewich et al., 2013). The secondary analysis was approved by the University of Michigan Institutional Review Board (HUM00153506).

Study protocols, including inclusion and exclusion criteria, as well as procedures have been previously described (Mozurkewich et al., 2013). Briefly, pregnant women at risk for depression based on a history of MDD or PPD or an EPDS score of 9–19 were recruited between 12 and 20 weeks’ gestation. Women meeting diagnostic criteria for MDD or who were taking antidepressant medications were excluded from the study, although participants who developed MDD during the study period were allowed to initiate antidepressant medications at the discretion of their physician. Following assessment of both inclusion and exclusion criteria, final determination of participant eligibility was made by administration of the Mini-Internal Neuropsychiatric Interview (MINI) by staff trained in clinical psychology (Sheehan et al., 1998). The MINI was used to exclude MDD as well as other psychiatric diagnoses including substance use or dependence. The primary outcome measure for the parent study was the Beck Depression Inventory Score at 6 weeks postpartum. The BDI was chosen as an instrument to assess depressive symptom severity based on prior studies of its use during pregnancy (Flynn et al., 2006; Marcus et al., 2011; Mozurkewich et al., 2013; Steer et al., 1990). For the current secondary analysis, we chose depression symptoms over the course of pregnancy, as measured by the BDI score, as the dependent variable of interest.

### 2.1. Participants

**Fig. 1.**
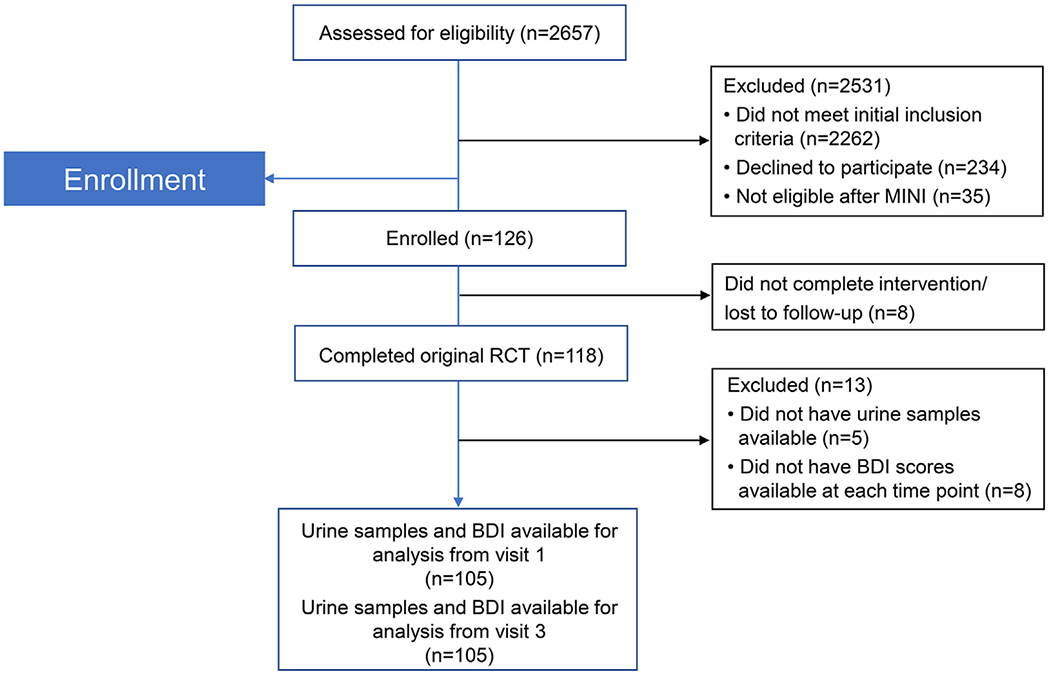
CONSORT diagram showing participant flow of original clinical trial MINI = Mini-International Neuropsychiatric Interviews BDI=Beck Depression Inventory.

**Fig. 2.**
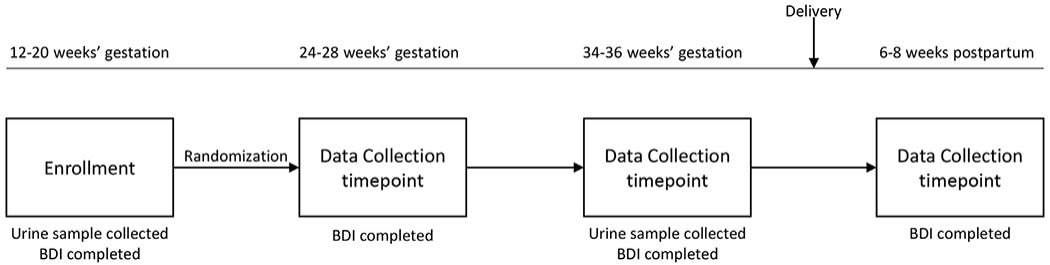
Schematic illustrating the “The Mothers, Omega-3 and Mental Health Study” BDI=Beck Depression Inventory.

The original study enrolled 126 women, 118 of whom completed the trial protocol. The participant flow is shown in Fig. 1. The enrolled participants were randomly assigned to receive EPA-rich fish oil, DHA-rich fish oil, or soy oil placebo. Maternal urine samples were collected at enrollment (12–20 weeks’ gestation) and in late pregnancy (34–36 weeks’ gestation). Participants completed the BDI scales at the time of enrollment, at 24–28 weeks’ and 34–36 weeks’ gestation, and at 6–8 weeks postpartum (Fig. 2). Of the original cohort, 105 participants completed all study visits and had urine samples available from both time points.

For the current investigation, we evaluated 49 urine metabolites in the stored maternal urine samples previously collected from the trial at 12–20 weeks’ gestation and 34–36 weeks’ gestation. The urinary metabolites analyzed in our study were chosen based on a combination of prior published research on urinary metabolites in depressive disorders (Lin et al., 2017; Zheng et al., 2013), as well as the types of assays that were available to run with the techniques developed in our metabolomics lab. The chosen assay was a glycolysis/trichloroacetic acid (TCA) assay, as it allowed for a greater number of metabolites to be evaluated than previously described, but also included several of the metabolites examined in prior studies.

### 2.2. Sample preparation

Urine microtubes were removed from −80 °C storage and maintained on wet ice throughout the processing steps. 100 μL of urine was added to a pre-chilled microtube and 0.4 mL of a mixture of methanol, chloroform, and water (8:1:1) containing isotope labeled internal standards (TCA-cycle analytes + creatinine-D3) was added. Microtubes were vortexed and allowed to incubate at 4 °C for 10 min to complete metabolite extraction. Samples were vortexed a second time, and then centrifuged at 12,000 RPM for 10 min at 4 °C. 100 μL of the extraction solvent was transferred to an autosampler vial for liquid chromatography-mass spectrometry (LC-MS) analysis. 10 μL of each sample was removed and pooled in a separate autosampler vial for quality control purposes. 280 μL of the extract was removed to an autosampler insert and dried under a stream of N2 at room temperature for 2.4 h. Prior to analysis, samples were reconstituted in 70 μL of a water/methanol mix (8:2) and vortex to aid resuspension.

### 2.3. Ion pairing reversed-phase LC-MS analysis

Analysis was performed on an Agilent system consisting of an Infinity Lab II ultra-performance liquid chromatography (UPLC) coupled with a 6545 QTof mass spectrometer (Agilent Technologies, Santa Clara, CA) using a JetStream electrospray ionization (ESI) source in negative mode. The following source parameters were used: Gas Temp: 250 °C; Gas Flow: 13 L/min; Nebulizer: 35 psi; Sheath Gas Temp: 325 °C; Sheath Gas Flow: 12 L/min; Capillary: 3500 V; Nozzle Voltage: 1500 V.

The UPLC was equipped with a 10-port valve configured to allow the column to be either eluted to the mass spectrometer or back-flushed to waste. The chromatographic separation was performed on an Agilent ZORBAX RRHD Extend 80 Å C18, 2.1 × 150 mm, 1.8 μm column with an Agilent ZORBAX SB-C8, 2.1 mm × 30 mm, 3.5 μm guard column. The column temperature was 35 °C. Mobile phase A consisted of 97:3 water/methanol and mobile phase B was 100% methanol; both A and B contained tributylamine and glacial acetic acid at concentrations of 10mM and 15mM, respectively. The column was back-flushed with mobile phase C (100% acetonitrile, no additives) between injections for column cleaning.

The LC gradient was as follows: 0–2 min, 0%B; 2–12 min, linear ramp to 99%B; 12–17.5 min, 99%B. At 17.5 min, the 10-port valve was switched to reverse flow (back-flush) through the column, and the solvent composition changed to 99%C. From 20.5 to 21 min, the flow rate was ramped to 0.8 mL/min, held until 22.5 min, then reduced to 0.6 mL/min. From 22.7 to 23.5 min, the solvent was ramped from 99% to 0%C while flow was simultaneously ramped down from 0.6 to 0.4 mL/min and held until 29.4 min, at which point flow rate was returned to starting conditions at 0.25 mL/min. The 10-port valve was returned to restore forward flow through the column at 28.5 min. An isocratic pump was used to introduce reference mass solution through the reference nebulizer for dynamic mass correction. Total run time was 30 min. The injection volume was 5 μL.

### 2.4. Metabolomic data analysis

Metabolites were identified by matching the retention time and mass (±10 ppm) to authentic standards. Peak areas were integrated using Profinder v8.00 (Agilent Technologies, Santa Clara, California). Data were normalized to urine creatinine levels and Loess drift correction, using Metabodrift 1.0 (Thonusin et al., 2017) and applied using the area of each metabolite in the control samples (pools) for correction.

### 2.5. Statistical analysis

#### 2.5.1. Outcome variables

Depressive symptoms were identified using established BDI cutoffs for scores less than or greater than those for mild depressive symptoms (BDI ≥9) (Beck et al., 1961, 1988). BDI scores were abstracted from the data as the primary endpoint by subtracting the initial (12–20 weeks′) BDI score from the 6–8 weeks’ postpartum BDI score (henceforth the “BDI score”). The Mood Trajectory throughout pregnancy was created as a categorical summary variable for clinically meaningful BDI scores. BDI scores were assigned corresponding Mood Trajectory labels: “Better,” “Same,” and “Worse.” These classifications were assigned to each pregnancy based on minimum clinically important differences (MCID) for BDI scores (Beck et al., 1961, 1988; Button et al., 2015; Masson and Tejani, 2013).

#### 2.5.2. Analytic processes

R statistical software (The R Foundation; Vienna, Austria) was used for analysis. Descriptive statistics were calculated for demographic and outcome variables to characterize the sample. Student T-tests and Pearson Product Moment Correlation Coefficients were used to examine the relationship between BDI score and participant race/ethnicity and age. For our primary analyses of metabolites, mixed model repeated measures analysis was used, with BDI score as the dependent variable. False discovery rate (FDR) correction was applied for multiple comparisons. Principal Component Analysis was used to evaluate metabolites and BDI scores across pregnancy and between participants. Urinary metabolites were assessed 1) at individual time points in pregnancy (12–20 weeks’ gestation and 34–36 weeks’ gestation) relative to BDI scores at those same time points; 2) vis-à-vis the summary variable of Mood Trajectory (“Better” vs “Same” vs “Worse”); and 3) in relation to BDI scores at 6–8 weeks’ postpartum. This exploratory approach was used to assess for data clusters and relationships that were not evident in the aforementioned mixed model repeated measures analysis.

Furthermore, given that the parent study randomized participants into EPA-rich fish oil, DHA-rich fish oil, and placebo groups, mixed model repeated measures analysis was performed to assess for any differences amongst metabolites in these groups as well.

## 3. Results

Of the 126 women from the original study cohort, 105 had urine samples and BDI scores available from each data collection time point and were included in the final analysis. Study subjects who missed the 34–36-week study visit due either to preterm delivery or missed appointment, or who previously withdrew from study participation, were not included in the final analysis.

**Fig. 3.**
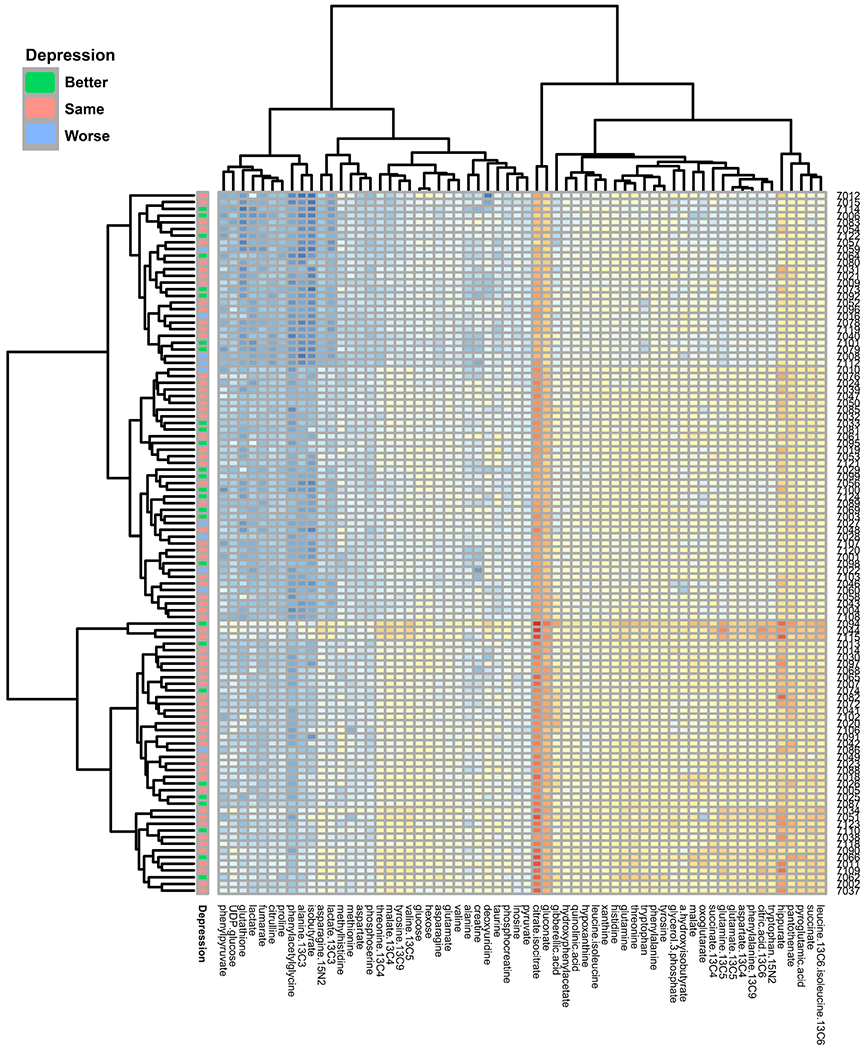
Heat map of mood trajectory Depressive symptoms grouped by log analyte (x-axis) concentration by participant (y-axis).

The mean age was 30.4 ± 5.3 years. The relationship between BDI scores and age was evaluated and found to be nonsignificant (*p* = 0.55). There were 87 white participants and 18 non-white participants. The relationship between BDI scores and race was evaluated and found to be nonsignificant (*p* = 0.49). Participant demographics are outlined in Table 1. The mean BDI score at 12–20 week′s gestation was 7.9 ± 5.4. Twenty-seven subjects were identified as having a “Better” Mood Trajectory, 10 subjects were identified as having a “Worse” Mood Trajectory, and 68 subjects were identified as having a “Same” Mood Trajectory (Fig. 3).

Individual metabolite levels, reported as area/nmol creatinine, are available in the Supplement.

For our primary analysis, we found that none of the 49 individual urinary metabolites (Table 2) examined at 12–20 weeks’ gestation and 34–36 weeks’ gestation were significantly associated with the BDI scores or the 6–8 weeks’ postpartum BDI assessment (Fig. 3).

However, increases in the urinary purine metabolites xanthine and hypoxanthine were associated with worsening Mood Trajectory (P-values of 0.03 and 0.02 respectively [Table 2, Fig. 3]). These associations did not retain statistical significance after FDR correction.

There were no significant differences in urinary metabolite concentrations between the randomized groups—EPA-rich fish oil, DHA-rich fish oil, and placebo.

## 4. Discussion

We found that increasing concentrations of the urine purine metabolites xanthine and hypoxanthine were associated with increasing depressive symptoms during pregnancy in our cohort. However, this relationship did not persist after FDR correction, possibly due to the limited sample size of this exploratory study. Thus, this preliminary finding should be interpreted with caution. Although not statistically significant after FDR correction, this finding supports the idea that there is likely a biological association between depressive symptoms and stress hormone-related urinary metabolites in women at risk for perinatal depression. The lack of additional correlates is reflective of the complexity of peripartum depressive symptoms and absence of a straightforward biologic marker. Furthermore, as discussed previously, peripartum depression may be a different entity neuro-hormonally than major depressive disorder given the influence of sex hormones and oxytocin that modulate the HPA axis response (Brummelte and Galea, 2010; Nazzari et al., 2020).

To our knowledge, this is the first study to demonstrate a potential associated between increased maternal urinary xanthine and hypoxanthine with increasing depressive symptoms in the pregnant population. Prior research in this area has demonstrated dysregulated purine metabolism, with altered concentrations of xanthine and hypoxanthine, in non-pregnant individuals with major depressive disorder (Agren et al., 1983; Ali-Sisto et al., 2016). However, these studies examined presence of the purine metabolites in serum and cerebral spinal fluid, not urine. Animal research using mouse depression models have shown changes in purine metabolites of the HPA axis, further supporting the findings of our study (Wu et al., 2017).

Depression is considered to be a state of oxidative stress (Black et al., 2015). Prior research has found that increased metabolic stress in depression can contribute to high risk of morbidity and mortality in those who suffer from depressive disorders (Black et al., 2015). Hypoxanthine is the precursor to xanthine, which is the precursor to uric acid—the end result of purine catabolism. The purine cycle contributes to the management of oxidative stress through the production of uric acid, which plays a role in lipid peroxidation (which in turn has a negative effect on nervous tissue), as well as production of superoxide radicals (Pritsos, 2000; Shichiri, 2014). To our knowledge, a role for purine cycle abnormalities in the pathogenesis of perinatal depression has not been previously described.

Identification of metabolic markers associated with perinatal depression could have significant clinical implications. These markers could be used as an objective measure in the recognition of perinatal depression, or of women who are at risk for perinatal depression—potentially allowing for earlier identification and intervention in those women who are at risk for depression complications associated with pregnancy.

Our study demonstrates that there is correlation with metabolic changes secondary to oxidative stress and depression given the increased levels of xanthine and hypoxanthine seen with increased depressive symptoms. There is need for future larger scale studies with greater statistical power to examine both the dysregulated purine metabolism pathway and oxidative stress in pregnancy. This would allow for more targeted research on the role of treatment and its effects on those in the pregnant population who suffer from peripartum depression.

Strengths of our study include the assessment of urinary metabolites at different time points throughout pregnancy. We analyzed a large number of different urinary metabolites, in addition to those that are a part of the purine degradation pathway. Furthermore, our urine samples came from a pregnant population that was found to be at high risk for depressive symptoms and postpartum depression, making it the optimal population to examine for aberrations in urinary metabolites related to depression in pregnancy. Finally, we were able to account for the influence of BMI in our sample analysis, as patient BMI is a known factor that can influence metabolite concentrations.

### 4.1. Limitations

There were several limitations to the study. First, our relatively small sample size limited the statistical power of our study to detect associations between urinary metabolites, depressive symptoms, and mood trajectory over time, which may account for the loss of significance in our findings after FDR correction. Similarly, this study was limited by its nature as an exploratory secondary biologic sample analysis from a trial whose sample size was determined by a different primary outcome and expected effect size.

Although current use of antidepressant medications was an exclusion criterion for participation in the original study, women in the trial were not prohibited from initiating antidepressant medications during the course of the study if clinically indicated. These medications may have altered metabolite concentrations in the urine of the minority of participants who initiated medications and may have likewise altered mood trajectory. Participants in the original study were randomized to receive either EPA- or DHA-rich fish oil, or soy oil placebo in pregnancy. However, our subgroup analyses demonstrated no differences in urinary metabolite between the randomized groups. Although we were able to account and assess for the influence of certain participant characteristics, such as BMI and fish oil supplementation, on our metabolite concentration, our sample size and study design did not allow for controlling of all potential factors that may influence urinary metabolite concentration. Finally, our sample population was homogenous, as a majority of participants were white.

## 5. Conclusions

This exploratory study found increasing concentrations of the urine purine metabolites xanthine and hypoxanthine to be associated with increasing depressive symptoms throughout the course of pregnancy. These metabolites may become potential objective targets for identifying women at risk for peripartum depressive symptoms and perinatal depression. Further studies are needed to establish a role for urinary metabolites in predicting depressive symptoms and mood trajectory during pregnancy and postpartum.

## Supplementary Material

1

## Figures and Tables

**Table 1:** Patient demographics (N = 105).

Parameter	Mean ± SD or n (%)
Age, years	30.4 ± 5.3
Race	
White	87 (82.6)
Black	9 (8.6)
Hispanic	5 (4.8)
Other	4 (3.8)
Parity	1.0 ± 1.0
Baseline BDI score	7.9 ± 5.4
Baseline BMI, kg/m^2^	27.6 ± 6.2

BDI=Beck Depression Inventory.

**Table 2:** Association of urinary metabolites with mood trajectory.

Metabolite	Change in BDI depressive symptom score^a^ relative to urinary metabolite concentration	
	Estimate	*p*-value
Alanine	−0.000787	0.32
Asparagine	0.000834	0.72
Aspartate	0.000578	0.31
Citrate + isocitrate	0.000643	0.75
Citrulline	0.000634	0.63
Creatine	−0.000392	0.37
Deoxyuridine	0.001070	0.85
Fumarate	0.000484	0.83
Gluconate	0.000390	0.83
Glucose	0.000064	0.65
Glutamate	0.000782	0.13
Glutamine	0.001088	0.98
Glutathione	0.001492	0.23
Glycerol 3-phosphate	0.001231	0.83
Hexose	0.000047	0.64
Histidine	0.000729	0.93
Hypoxanthine	0.000828	0.02^b^◆
Inosine	0.000033	0.22
Lactate	0.000961	0.98
Leucine + Isoleucine	0.000349	0.65
Malate	−0.000204	0.71
Methionine	0.000265	0.76
Ornithine	0.001238	0.54
Oxoglutarate	0.001473	0.61
Pantothenate	0.000964	0.26
Phenylalanine	0.000666	0.19
Phenylpyruvate	−0.000257	0.16
Phosphocreatine	0.000211	0.61
Phosphoserine	0.000688	0.21
Proline	0.000129	0.89
Pyruvate	0.000492	0.96
Succinate	−0.000099	0.51
Taurine	0.001840	0.61
Threonine	0.000136	0.73
Tryptophan	0.001075	0.58
Tyrosine	0.000287	0.70
UDP-glucose	0.001258	0.96
Valine	0.000621	0.93
Xanthine	0.000969	0.03^b^◆
Hydroxyphenylacetate	0.000565	0.32
Methylhistidine	−0.000177	0.07
Hippurate	0.000155	0.70
Pyroglutamic acid	0.000647	0.58
Quinolinic acid	0.000549	0.87
Isobutyrate	0.001635	0.55
Phenylacetylglycine	0.000239	0.06
Hydroxyisobutyrate - A	0.000489	0.51
Hydroxyisobutyrate - B	0.000085	0.45
Hydroxyisobutyrate - C	0.000286	0.16

BDI=Beck Depression Inventory; FDR = false discovery rate.

◆ increasing concentration of metabolite with worsening mood trajectory.

a From 12–20 weeks′ gestation to 34–36 weeks’ gestation.

b Statistically significant before FDR correction applied.

## Abbreviations:

MDD: Major Depressive Disorder
PPD: Postpartum depression
HPA: Hypothalamic pituitary adrenal
EPDS: Edinburgh Postnatal Depression Scale
BDI: Beck Depression Inventory
MINI: Mini-Internal Neuropsychiatric Interview
EPA: eicosapentaenoic acid
DHA: docosahexaenoic acid
TCA: trichloroacetic acid
LC-MS: liquid chromatography-mass spectrometry
UPLC: ultra-performance liquid chromatography
FDR: false discovery rate
